# Transjugular intrahepatic portosystemic shunt placement: portal vein puncture guided by 3D/2D image registration of contrast-enhanced multi-detector computed tomography and fluoroscopy

**DOI:** 10.1007/s00261-020-02589-1

**Published:** 2020-05-25

**Authors:** Timo C. Meine, Cornelia L. A. Dewald, L. S. Becker, Aline Mähringer-Kunz, Benjamin Massoumy, Sabine K. Maschke, Martha M. Kirstein, Thomas Werncke, Frank K. Wacker, Bernhard C. Meyer, Jan B. Hinrichs

**Affiliations:** 1 Department of Diagnostic and Interventional Radiology, Member of the German Center for Lung Research (DZL), Hannover Medical School, Hannover, Germany; 2 Department of Diagnostic and Interventional Radiology, Johannes Gutenberg-University Medical Centre, Mainz, Germany; 3 Department of Gastroenterology, Hepatology and Endocrinology, Hannover Medical School, Hannover, Germany; 4 Institute for Diagnostic and Interventional Radiology, Hannover Medical School, Carl-Neuberg-Str. 1, 30625 Hannover, Germany

**Keywords:** Transjugular intrahepatic portosystemic shunt, Image-guided therapy, Venous intervention, Image registration, Operator experience

## Abstract

**Background:**

To assess the technical feasibility, success rate, puncture complications and procedural characteristics of transjugular intrahepatic portosystemic shunt (TIPS) placement using a three-dimensional vascular map (3D-VM) overlay based on image registration of pre-procedural contrast-enhanced (CE) multi-detector computed tomography (MDCT) for portal vein puncture guidance.

**Materials and methods:**

Overall, 27 consecutive patients (59 ± 9 years, 18male) with portal hypertension undergoing elective TIPS procedure were included. TIPS was guided by CE-MDCT overlay after image registration based on fluoroscopic images. A 3D-VM of the hepatic veins and the portal vein was created based on the pre-procedural CE-MDCT and superimposed on fluoroscopy in real-time. Procedural characteristics as well as hepatic vein catheterization time (HVCT), puncture time (PT), overall procedural time (OPT), fluoroscopy time (FT) and the dose area product (DAP) were evaluated. Thereafter, HVCT, PT, OPT and FT using 3D-VM (61 ± 9 years, 14male) were compared to a previous using classical fluoroscopic guidance (53 ± 9 years, 21male) for two interventional radiologist with less than 3 years of experience in TIPS placement.

**Results:**

All TIPS procedure using of 3D/2D image registered 3D-VM were successful with a significant reduction of the PSG (*p* < 0.0001). No clinical significant complication occurred. HVCT was 14 ± 11 min, PT was 14 ± 6 min, OPT was 64 ± 29 min, FT was 21 ± 12 min and DAP was 107.48 ± 93.84 Gy cm^2^. HVCT, OPT and FT of the interventionalist with less TIPS experience using 3D/2D image registered 3D-VM were statistically different to an interventionalist with similar experience using fluoroscopic guidance (*p*_HVCT_ = 0.0022; *p*_OPT_ = 0.0097; *p*_FT_ = 0.0009). PT between these interventionalists was not significantly different (*p*_PT_ = 0.2905).

**Conclusion:**

TIPS placement applying registration-based CE-MDCT vessel information for puncture guidance is feasible and safe. It has the potential to improve hepatic vein catherization, portal vein puncture and radiation exposure.

## Introduction

Transjugular intrahepatic portosystemic shunt (TIPS) is a highly effective standard procedure to reduce the portosystemic pressure gradient in patients suffering from complications of portal hypertension such as ascites and variceal bleeding [1–7]. The most challenging and a time-consuming steps during the TIPS procedures are the catheterization of the appropriate hepatic vein and the puncture of the portal vein under fluoroscopic guidance. Technical complications during TIPS procedures are associated with off-target punctures causing injury of the liver arteries and the biliary tract or perforation of the liver capsule potentially leading to arterioportal or biliary-portal fistulas, subcapsular hematoma or life-threatening intraperitoneal hemorrhage [1, 8, 9]. Moreover, a suboptimal position of the TIPS stent is associated with an increased risk for shunt dysfunction [10]. Thus, the interventionalist has to estimate the optimal needle path on pre-interventional acquired three-dimensional (3D) contrast-enhanced (CE) multi-detector computed tomography (MDCT) or magnetic resonance imaging (MRI) [11, 12]. Several guidance techniques have been reported to facilitate the portal vein puncture aiming to reduce puncture times and peri-procedural complications, which are usually affected by the experience of the interventionalist [11, 13]. Blind fluoroscopic puncture can be augmented with direct or indirect 2D visualization of the portal vein by wedged portography or transsplenic/transarterial mesenteric portography [14, 15]. The portal vein puncture can also be effectively guided by ultrasound [16]. However, an experienced sonographeur has to be involved in addition to the operator placing the TIPS. More recently, several 3D guidance techniques for TIPS placement have been reported [12, 17–24]. Rouabah et al. described a guidance technique based on a 3D volume rendering of the portal vein generated from a contrast-enhanced MDCT Angiography (MDCTA) of the portal trunk acquired shortly before the TIPS, which was superimposed on the fluoroscopic images [19]. Tacher et al. and Luo et al. acquired an unenhanced native CACT (C-Arm Computed Tomography) during the TIPS procedure and co-registered this with a previously acquired CE-MDCT to visualize the portal vein on fluoroscopy [20, 23]. In another study, Luo et al. introduced a guidance technique relying on intra-procedural acquisition of a CE-CACT with contrast injection in the superior mesenteric artery to obtain an indirect portomesentericography serving as an overlay [24]. Comparable to this technique are the CE-CACT-assisted guidance techniques published by Chivot et al. and Ketelsen et al. [12, 22]. Chivot et al. used an indirect wedge portography during CACT acquisition [22] and Ketelsen et al. administered the contrast media via an intravenous access and acquired the CACT in portal venous phase [12]. Another group reported on the co-registration of CE-MDCT with an unenhanced CACT and the CE-CACT with intravenous contrast injection for portal vein puncture guidance [21]. All of these techniques aim to visualize the portal vein during the intervention with a varying complexity in displaying the portalvenous anatomy and most of them need to acquire additional 3D datasets (CACT, CE-CACT or MDCTA) causing additional radiation exposure. Using the vessel information of both the hepatic and the portal veins of a CE-MDCT acquired during standard TIPS work-up might improve the portal vein puncture guidance during the intervention through a fluoroscopic registration approach. Furthermore, this might have the potential to offer a 3D guiding opportunity without the need to acquire a new (CE-) CACT or MDCTA. Therefore, the purpose of our study was to investigate the procedural characteristics, the technical success and the puncture-related complications of TIPS placement using a 3D/2D image registration of hepatic and portal veins segmented on a previously acquired CE-MDCT with two fluoroscopic images for guidance of the portal vein puncture focused on low-experienced interventionalists.

## Materials and methods

### Study population

Between 11/2018 and 06/2019 thirty-eight TIPS procedures were retrospectively reviewed in our institutional Picture Archiving Computer System (PACS) for study inclusion. Inclusion criteria were elective TIPS procedures in patients with refractory portal hypertension older than 18 years of age. Emergency and transsplenic TIPS placements were excluded. Overall, twenty-seven patients with portal hypertension (59 ± 9 years, 18 male and 9 female) who received elective TIPS placement using 3D/2D image registration for procedure guidance were included in the study. The data of the interventionalist with limited experience (< 3 years) in TIPS procedures using 3D/2D image registration were compared to data from a classical fluoroscopic guided TIPS study cohort at our hospital [11]. Out of this study, including 102 TIPS placements 40 consecutive procedures were performed by an interventionalist with a level of experience less than three years. After exclusion of six emergency TIPS placements and four TIPS placements with incompleted data documentation 30 were included in our study for comparison. Written informed consent was obtained for all patients. Our institution’s Human Subjects Research Review Board approved our retrospective study, and a waiver for informed consent was given.

### Preinterventional workup

Clinical examination and laboratory tests (blood and coagulation panel, serum electrolyte levels, thyroid-stimulating hormone level, creatinine level and liver function tests) were performed to exclude presence of contraindications for TIPS placement [25, 26]. An evacuating ascites puncture was performed routinely before the intervention. No blood products needed to be administered pre-, intra- or post-interventionally. Doppler ultrasound of both internal jugular veins was performed to exclude jugular vein thrombosis. Conventional CE-MDCT of the liver was acquired to assess the liver anatomy and to exclude portal vein thrombosis, dilation of the bile ducts and liver malignancies. The CT images were generated on a 64-row MDCT (GE Lightspeed VCT, GE Healthcare, Chalfont St. Giles, United Kingdom) (detector collimation: 64 × 0.625 mm, slice thickness: 1.25 mm, interval: 1 mm) or on a dual source 2 × 96-row MDCT (Somatom FORCE, Siemens Healthineers, Forchheim, Germany) (detector collimation: 192 × 0.7 mm, slice thickness: 1 mm, intevall: 0.7 mm). The whole abdomen was scanned during a single breathhold. The contrast medium was administered by a mechanical injector through an appropriate peripheral venous access. Arterial and portovenous contrast phase were acquired according to Choi et al. [27, 28]. In one case a CE-MDCT acquired in an external hospital was used for image registration and guidance. Median time between TIPS and CE-MDCTs was 13 days.

### Image registration

**Fig. 1:**
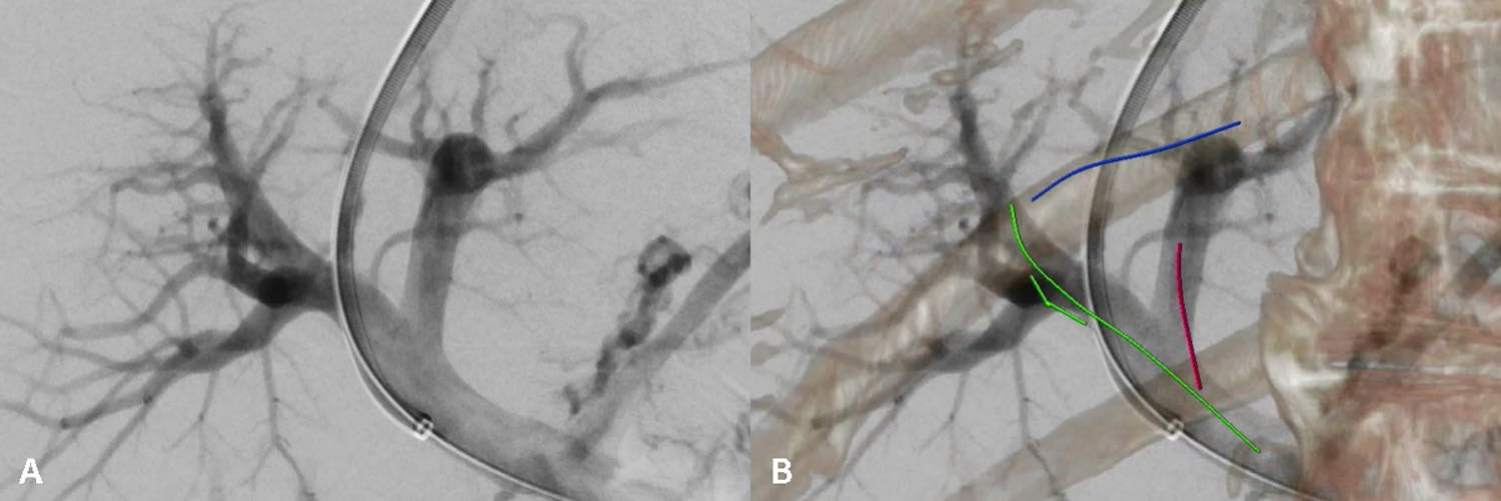
3D/2D image registered 3D-VM. In A the direct portography with the TIPS sheath in the main portal trunk after successful portal vein puncture is shown. In B the 3D-VM is illustrated with the right hepatic vein indicated in blue, the main portal trunk and the right portal vein in green and the left portal vein in red. Vertebral bodies and rips were superimposed to confirm the alignment during the procedure. Note: Both images are displayed in the angio suite during the intervention in real-time. Abbreviations: 2D = two-dimensional, 3D = three-dimensional, 3D-VM = three-dimensional vascular map

DICOM-Images of the pre-procedural CE-MDCT were uploaded and postprocessed on the angiographic workstation using dedicated software in the forefront of the procedure (syngo X Workplace^®^ VD20D Siemens Healthcare, Germany). In order to generate a three-dimensional vascular map (3D-VM) of the hepatic veins and portal veins (Fig. 1), the interventional radiologist labeled the central part of the hepatic veins, the main portal vein trunk and the left and right portal vein branches manually on the portalvenous phase CE-MDCT using a centerline segmentation tool. This approach is comparable to and has been described in detail for image guidance of balloon pulmonary angioplasties [29]. With the patient on the table, the interventional radiologist acquired fluoroscopic images of the abdomen in frontal and lateral view followed by an automatic 3D/2D image registration with the previously generated 3D-VM. If necessary, manual adjustment using landmarks such as vertebral bodies, ribs, pelvis or gallbladder stones was applied. The registration process is typically done after successful puncturing of the jugular vein and the pressure measurements in the inferior caval vein. Finally, the 3D-VM was automatically overlaid onto real-time fluoroscopy and displayed in the angio room. Since the 3D-VM overlay is registered, it follows any table movement, C-Arm angulation and image zoom applied during the procedure, providing a precise 3D vascular overlay of the portal vein branches and the hepatic veins for puncture guidance in real-time (Fig. 1).

### TIPS procedure

All TIPS procedures were conducted under general anesthesia on the Siemens Artis Q^®^ (*n* = 7) or Siemens Artis Pheno^®^ (*n* = 20) angiography system equipped with the same software (Syngo^®^ Siemens Healthcare, Germany). TIPS were established by two interventional radiologists (*n* = 22 by J. B. H. with < 3 years experience and *n* = 5 by B. C. M. with > 5 years experience in TIPS placement). Venous access was achieved by ultrasound-guided puncture of the right internal jugular vein. Afterwards, the interventionalist inserted a 10-French introducer and placed the TIPS needle (GORE TIPS Set, Gore, AZ, USA) in the right hepatic vein. A wedged portography was acquired according to our standard operating procedures. Under 3D/2D image registered 3D-VM the puncture with the TIPS needle was guided from the right hepatic vein into the right portal vein branch. Successful portal vein puncture was verified by injection of contrast agent. Thereafter, a 0.035-inch guidewire was introduced into the portal vein and the puncture tract was dilated with an 8-mm balloon catheter (Mustang, Boston Scientific, MA, USA). Finally, a TIPS stent graft (Viatorr, Gore, AZ, USA) was placed connecting the right hepatic vein and the right portal vein branch. The diameter of the self-expandable stent graft was initially set to 8 mm. A control portography with a pigtail catheter in the main portal vein was performed using a standardized contrast injection protocol. The pressure in the inferior vena cava (IVC) and the portal venous pressure (PVP) were measured and the portosystemic gradient (PSG) was calculated before and after TIPS placement. If the PSG was > 12 mmHg the TIPS was dilated to 10 mm diameter to reach a sufficient reduction of the PSG [11]. Additional variceal embolization was performed using Vascular Plugs (Amplatzer Vascular Plug, AGA Medical Corporation, MN, USA) when the patient had history of variceal bleeding. After the TIPS placement, the patients were monitored for at least 24 h at the intensive care unit and were transferred within 48 h to the general ward. Puncture-related clinical significant complications such as subcapsular hematoma, intraperitoneal hemorrhage and death were assessed with fluoroscopic imaging during the intervention or with clinical examination, abdominal ultrasound and blood tests on intensive care unit within 48 h after TIPS placement. Safety data were collected from the medical transfer reports or medical discharge reports.

### Definitions and data collection

Patient’s demographics included age, sex, body-mass-index (BMI), primary disease, model of endstaged liver disease (MELD) score and indication for TIPS placement. Indications were refractory gastrointestinal bleeding and refractory ascites or hydrothorax according to the European Association of the Study of the Liver (EASL) Clinical Practice Guidelines 2018 [26]. Furthermore, additional procedures like placement of a second stent for TIPS extension or variceal embolization in the history of bleeding were recorded. Technical success was defined as a portosystemic gradient (PSG) of equal or less than 8 mmHg after TIPS placement [11, 20, 23, 24].

Clinical significant complication of TIPS procedures were defined according major complication of the Proposal of a New Adverse Event Classification by the Society of Interventional Radiology Standards of Practice Committee, which require therapy with hospitalization of < 48 h or > 48 h and could lead to permanent adverse sequelae and/or death [30].

The hepatic vein catherization time (HVCT) was defined as the time from jugular vein puncture to successful catherization of the appropriate hepatic vein, which is confirmed by the following acquisition of the wedged portography. The puncture time (PT) was defined as the time from the wedged portography to the first documented image with the established guidewire or catheter in the portal vein branch. The overall procedural time (OPT) was defined as time from jugular vein puncture to removal of the sheath. The fluoroscopy time (FT) is the total time needed to acquire the fluoroscopic images. Moreover, the number of Digital Subtraction Angiography (DSA) series and the dose area product (DAP) were recorded. Furthermore, HVCT, PT, OPT and FT of the interventional radiologist with limited experience (< 3 years) in TIPS procedures of this study were compared to data acquired during a previous TIPS study at our hospital without 3D/2D image registered 3D-VM [11]. In that study, TIPS placements were performed using fluoroscopic guidance on a MultiDiagnost 4 with DSI-Angio and Easy-Vision software, but no CACT function was available (2000, Philips Healthcare, The Netherlands) [11].

### Statistical analysis

Statistical analysis was performed using R 3.6.2 statistical computation system (https://www.r-project.org). Median and interquartile ranges were given for categorical data. Continuous variables were shown with mean and standard deviation (SD). Parameter changes following TIPS placement were analyzed using the two-sided Wilcoxon-signed rank test. Comparison between interventionalists with limited experience performing TIPS placements either with 3D/2D image registered 3D-VM or with classical fluoroscopic guidance were analyzed using two-sided Mann–Whitney-U test for continuous variables and with the Fisher’s exact test for category variables. Level of significance was *p* < 0.05.

## Results

All 27 patients (59 ± 9 years, 18 men, 9 women) underwent successful TIPS placement using 3D/2D image registration of pre-procedural CE-MDCT vessel information with intra-procedural fluoroscopy for puncture guidance. The causes of portal hypertension were Budd-Chiari syndrome (3/27) and cirrhosis due to ethyltoxic liver disease (11/27), non-ethyltoxic steatohepatitis (3/27), hepatitis B (1/27), hepatitis C (2/27), hemochromatosis (1/27), re-cirrhosis after orthotopic liver transplantation (2/27) and cryptogenic cirrhosis (4/27). The indications for TIPS were recurrent or refractory variceal or gastrointestinal bleeding (6/27) and refractory ascites or hydrothorax (21/27). Additional procedures were performed in 10 patients with placement of a second stent graft to extend the previously placed stent graft in two patients and variceal embolization in eight patients. Technical success was achieved in all patients with the significant reduction of the PSG (15 ± 3 mmHg to 4 ± 3 mmHg; *p* < 0.0001). No clinical significant complication occurred and no patient died because of TIPS placement. Data are summarized in Table 1. The procedural characteristics included a mean HVCT of 14 ± 11 min, a PT of 14 ± 6 min and an OPT of 64 ± 29 min. A FT of 21 ± 12 min was recorded. The median number of DSA series was 4 with a range from 3 to 6. The mean overall DAP was 107.48 ± 93.84 Gy cm^2^. For details refer to Table 2.

**Table 1:** Patient’s characteristics

Patient’s Characteristics	
Age (years)	59 ± 9
Sex (male/female)	18/9
BMI (kg/m^2^)	24 ± 6
MELD score	11 ± 5
Liver disease	
Ethyltoxic	11
HBV	1
HCV	2
NASH	3
BC	3
HC	1
Post-LTx	2
Cryptogenic	4
Indication	
Refractory variceal or gastrointestinal bleeding	6
Refractory ascites and hydrothorax	21
Clinical significant complication	
Subcapsular hematoma	0
Intraperitoneal hemorrhage	0
Death	0
Additional procedures	
Second stent placement	2
Variceal embolization	8
Technical success	
Success rate	27/27
Preprocedural PSG (mmHg)	15 ± 3
Postprocedural PSG (mmHg)	4 ± 3

Sex, age, body-mass-index (BMI), model of endstaged liver disease (MELD) score, liver disease, indication, clinical significant complication, additional procedures and technical success were tabulated

*BC* Budd-Chiari syndrome, *HBV* hepatitis virus B, *HC* hemochromatosis, *HCC* hepatocellular carcinoma, *HCV* hepatitis virus C, *NASH* non-alcoholic steatohepatitis, *post*-*LTx* cirrhosis after orthotopic liver transplantation, *PSG* portosystemic gradient

**Table 2:** Procedural characteristics and comparison to literature

Procedural characteristics	Study group	Böning et al.	Ketelsen et al.	Luo et al.‘18	Luo et al.‘17	Rouabah et al.
Planning modality	CE-MDCT	CE-MDCT or CE-CACT	CE-CACT	CE-CACT	CE-MDCT	MDCTA
Vascular label	hv, pvb	pvb, vnp	pvb	pvb	hv, pvb	pvb
Image registration technique	CE-MDCT on FS	CE-MDCT/CACT on FS or CE-CACT on FS	CE-CACT on FS	CE-CACT on FS	CE-MDCT/CACT on FS	MDCTA on FS
	3D/2D	3D/3D or 3D	3D	3D	3D/3D	3D/2D
Number of patients	27	21	12	20	15	18
Hepatic vein catherization time (min)	14 ± 11	n.a.	n.a.	n.a.	n.a.	n.a.
Puncture time (min)	14 ± 6	32 ± 45	14 ± 8	n.a.	n.a.	17 ± 9
Overall procedure time (min)	64 ± 29	115 ± 52	66 ± 29	n.a.	60 ± 13	n.a.
Fluoroscopy time (min)	21 ± 12	n.a.	18 ± 9	11 ± 2	14 ± 4	n.a.
Number of DSA series	4, 3–6	n.a.	17.5, 15.25–23.5	n.a.	n.a.	n.a.
Dose area product (Gy cm^2^)	107.48 ± 93.84	563.00 ± 289.00	188.16 ± 121.18	295.50 ± 66.60	152.11 ± 86.63	258.53 ± 161.41

Image registration technique, number of patients, overall procedure time, fluoroscopy time, number of DSA series and Dose-Area-Product were tabulated for the study population and the literature. Times were shown as median ± standard deviation. Number of Digital Substraction Angiography (DSA) series were tabulated as median and range (minimum-maximum)

*2D* two-dimensional, *3D* three-dimensional, *CE* contrast enhanced, *CACT* C-arm-computed tomography, *MDCT* multi-detector-computed tomography, *MDCTA* contrast-enhanced multi-detector-computed tomographic angiography, *n.a.* not available, *FS* fluoroscopy, *hv* hepatic veins, *pvb* portal vein branches, *vnp* virtual needle path (from the catheter in the hepatic vein to the portal vein branch). Data of the publication by Tacher et al. and Chivot et al. are mentioned in the text, because they reported median and range

Comparing the study groups of the interventionalists with limited level of experience in TIPS placement of less than 3 years using 3D/2D registered 3D-VM (61 ± 9 years, 14 men, 8 women) or classical fluoroscopic guidance (53 ± 9 years, 21 men, 9 women), only the patient’s age was significant higher in the current study group than in the previously published cohort (*p* = 0.0030). Sex, BMI, primary disease, MELD score, indiciation, clinical significant complication and the amount of additional procedures were not statistically different (*p*_sex_ = 0.7665, *p*_BMI_= 0.5651, p_MELD_ = 0.8892, *p*_primary disease_ = 1, *p*_indication_ = 0.7411, *p*_clinical significant complication_ = 1, *p*_additional stent_ = 0.4996, *p*_variceal embolization_ = 0.0789). Technical success was achieved in all patients with the significant reduction of the PSG under 3D/2D registered 3D-VM (27/27; 16 ± 4 mmHg to 4 ± 3 mmHg; *p* = 0.0419) and in 29/30 patients under classical fluoroscopic guidance (15 ± 5 mmHg to 6 ± 3 mmHg; *p* = 0.0026). The success rate was not statistically different between interventionalists with 3D/2D registered 3D-VM and classical fluoroscopic guidance (p_success rate_ = 1). HVCT, OPT and FT were shorter in the 3D/2D registered 3D-VM group performed by an interventionalist with less than three years of TIPS experience (12 ± 7 min vs.28 ± 18 min, *p*_HVCT_ = 0.0022; 53 ± 13 min vs. 85 ± 27 min, *p*_OPT_ = 0.0097; 16 ± 7 min vs. 27 ± 12 min; *p*_FT_ = 0.0009). However, the PT between these interventionalists was not significantly different (13 ± 6 min versus 19 ± 15 min; *p*_PT_ = 0.2905); for details refer to Table 3.

**Table 3:** Comparison of patient’s and procedural characteristics between interventionalists with limited experience

Patient’s Characteristics	3D/2D	CFS	*p* value
Age (years)	61 ± 9	53 ± 9	0.0030
Sex (male/female)	14/8	21/9	0.7665
BMI (kg/m^2^)	25 ± 7	25 ± 5	0.5651
MELD score	11 ± 4	11 ± 6	0.8892
Liver disease (ethyltoxic/non-ethyltoxic)	9/13	13/17	1
Indication (ref. bleeding/ref. ascites and hydrothorax)	4/18	7/23	0.7411
Clinical significant complication	0	0	1
Additional procedures			
Second stent placement	0	2	0.4996
Variceal embolization	7	3	0.0789
Technical success			
Success rate	22	29	1
Preprocedural PSG (mmHg)	16 ± 4	15 ± 5	0.2887
Postprocedural PSG (mmHg)	4 ± 3	6 ± 3	0.0356
Procedural characteristics	3D/2D	CFS	*p* value
Hepatic vein catheterization time (min)	12 ± 7	28 ± 18	0.0022
Puncture time (min)	13 ± 6	19 ± 15	0.2905
Overall procedure time (min)	53 ± 13	85 ± 27	0.0097
Fluoroscopy time (min)	16 ± 7	27 ± 12	0.0009

Patient’s and procedural characteristics for the study populations of the interventionalists with limited experience using 3D/2D image registered 3D-VM (3D/2D) and classical fluoroscopic guidance (CFS) were compared. Patient’s characteristics included sex, age, body-mass-index (BMI), model of endstaged liver disease (MELD) score, liver disease, indication, clinical significant complication, additional procedures and technical success were tabulated. Procedural characteristics were hepatic vein catherization time, overall procedure time and fluoroscopy time. Values for TIPS placement with classical fluoroscopic guidance were obtained from a previous cohort of our publication by Marquardt et al. [11]. Continuous variables were shown as mean ± standard deviation. *p* values were reported for two-sided Mann–Whitney-U test for continuous variables or two-sided Fischer’s exact test for category variables between interventionalists with limited experience performing TIPS placements with 3D/2D image registered 3D-VM and classical fluoroscopic guidance

*2D* two-dimensional, *3D* three-dimensional, *3D*-*VM* three-dimensional vascular map, *PSG* portosystemic gradient, *ref. bleeding* refractory variceal or gastrointestinal bleeding, *ref. ascites and hydrothorax* refractory ascites and hydrothorax

## Discussion

3D/2D image registration combining CE-MDCT vessel information with real-time fluoroscopy for guidance of the portal vein puncture is feasible and safe. Our 3D/2D image registration technique superimposes 3D information of the hepatic and portal veins as a 3D-VM on real-time fluoroscopy during the intervention. Therefore, 3D/2D image registered 3D-VM facilitates catheterization of the appropriate hepatic vein followed by successful 3D-guided puncture the portal vein, the most challenging step of TIPS procedures.

The demographics of our study population and the indications for TIPS placement are comparable to the recent literature on 3D-guided TIPS procedures [12, 19–22] except for the studies by Luo et al., who included younger patients with refractory bleeding [23, 24]. In our study, the TIPS placement under guidance of the 3D/2D image registered 3D-VM was successfully performed in all cases with a significant reduction of the PSG. Moreover, no clinical significant complication occurred. This reflects the good safety profile of our approach and is in line with recent reports on guidance techniques for TIPS placement [12, 19–21, 23, 24].

Reviewing the procedural characteristics, the overall mean PT of 14 ± 6 min and OPT of 64 ± 29 min in our study are short compared to the described studies on image-guided TIPS placement with PTs ranging from 14 min to 32 min [12, 19, 21, 22] and OPTs ranging from 40 min to 115 min (s. Table 2) [20–22]. Most of the other studies on guidance techniques did not include additional procedures like variceal embolization or second stent graft placements potentially prolonging the OPT of our study (s. Table 1) [19–22]. A major reason for the longer OPTs in the previous studies without performing additional procedures might be the time needed for the acquisition of the additional CACT and the quite complex post-processing/reconstruction of the image fusion algorithms used in these studies. For example, Luo et al. performed an additional arterial puncture and catheterization of the superior mesenteric artery for the acquisition of a CE-CACT during an indirect portography [24]. Rouabah et al. needed a relatively long post-processing time for a complex 3D-reconstruction of the portal trunk from an additionally acquired MDCTA [19]. Of note, the MDCTA in the study by Rouabah et al. was additionally acquired for the purpose of portal vein segmentation and overlay. Both studies reported on an average of 15 min for “3D-overlay-readiness” [19, 24], which obviously prolonged their TIPS procedure. In our study, the generation of the centerlines within the hepatic vein and the portal vein on the portalvenous CE-MCDT, the acquisition of two fluoroscopic images in perpendicular views and the superimposition of the 3D-VM on the fluoroscopy takes approximately 3-5 min. The overall mean and median DAP in our study were 107.48 Gy cm^2^ and 61.14 Gy cm^2^. This is clearly less than the most recent reference DAP of 446.00 Gy cm^2^ for TIPS procedures in the U.S. [31]. In detail, the DAP in our study is 30-80% lower than the mean DAPs reported in five studies using registration-guided TIPS procedures that range from 144.2 to 563.00 Gy cm^2^ [12, 19–24]  (s. Table 2). The difference between our median DAP of 61.14 Gy cm^2^ to the lowest DAP for CACT-assisted TIPS procedures of 90.75 Gy cm^2^ reported by Tacher et al. might best be explained with the additional radiation exposure of the CACT reported to range from 18.00 to 63.90 Gy cm^2^ [12, 21, 23]. Moreover, Tacher et al. reported on CACT acquisition with the patient’s arms placed next to the body [20]. This technique might be associated with additional radiation exposure and might be counterproductive for the accuracy of the co-registration with a CE-MDCT, which is typically acquired with the patients’s arms up.

Focusing on the interventionalist with limited experience in TIPS procedures, our mean PT of 13 min and OPT of 53 min were 30% faster using 3D/2D image registered 3D-VM compared to the reported guidance techniques in the literature (s. Table 2). This finding is underlined by the comparison of the interventionalists with limited experience performing TIPS at our hospital. We found a significant difference with a 55% lower HVCT, 35% lower OPT and 40% lower FT when using 3D/2D image registered 3D-VM. The mean PT of the interventionalist with a level of experience of less than three years using 3D/2D image registered 3D-VM was 30% shorter than the mean PT using classical fluoroscopic guidance, but the difference yielded no statistical significance. Since Marquardt et al. showed a correlation between PT and OPT [11], the short OPT in our study might at least partly be explained by the trend of a shorter PT. Another possible explanation for the short OPT using 3D/2D image registered 3D-VM might be the additional labeling of the hepatic veins, which significantly improves the catheterization time of the appropriate hepatic vein and might also support stent positioning. This might be of additional value, especially in patients with altered anatomy due to liver cirrhosis or after liver surgery or orthotopic transplantation. Of note, after successful catheterization of the right hepatic vein, it is possible to re-evaluate and to re-align a potential 3D-VM off-set using control-elements at the table-side in the angiography suite. Thus, labeling of the hepatic veins offers additional guidance which can save fluoroscopy time and radiation exposure at the beginning of the TIPS procedure when compared to approaches which require successful catheterization of the right hepatic vein prior to CACT acquisition to determine the starting point of the puncture guidance [20–22].

In comparison to the published image guidance techniques the 3D/2D image registered 3D-VM presented here has additional advantages. By labeling the hepatic veins and the portal vein branches for a 3D-VM this approach differs from recently published image guidance techniques which are mainly focused on the portal vein [12, 19–22, 24]. Labeling both, entry and target point as well as the distance to each other illustrates the two most important informations for puncture procedure in one real-time image/overlay. The combination of the centerline drawings in the hepatic veins and the portal vein in our 3D-VM might be more robust to changes caused by patient breathing during the procedure or paracentesis following the acquisition of the CE-MDCT. Such changes of the liver position due to paracentesis were the reason why Rouabah et al. performed an additional MDCTA of the portal trunk after patients underwent paracentesis [19]. A common approach to confirm the needle position in the appropriate hepatic vein and to visualize the portal vein branch is a wedged portography. However, this requires an additional DSA run and can be associated with severe subcapsular hematomas as reported by Chivot et al. [9, 22]. The technique to overlay both, hepatic and portal veins, renders wedged portographies or additional CACT, CE-CACT or MDCTA unnecessary. Therefore, it not only shortens the procedure workflow significantly but also reduces the potential for complications. Nevertheless, we performed wedge portographies in our study according to our standard operating procedures.

Overall, our 3D/2D image registered 3D-VM is a unique approach in using a CE-MDCT acquired during routine patient workup and planning of the TIPS placement and two fluoroscopic images in perpendicular views without the need to acquire an additional 3D dataset [12, 19–24]. The 3D-VM provides robust information about the localization of the hepatic veins in relation to the portal vein branches, thereby displaying entry and target for a successful puncture. Our 3D/2D image registered 3D-VM seems to be beneficial for interventionalists with a low level of TIPS experience compared to classical fluoroscopic guidance and improves patient safety due to expendable wedged portography and reduces fluoroscopy and procedure time. We believe that image guidance techniques are helpful to support the training of interventional radiologists not only for TIPS placement but also for other interventional procedures (e.g., balloon pulmonary angioplasty, transarterial chemoembolization or prostatic artery embolization [29, 32, 33]. In addition, the respective handling- and software-skills are useful to facilitate even more complex image-guided interventions such as fenestrated endovascular aneurysm repair [34].

### Limitations

One major limitation is the retrospective nature of our study. Puncture attempts are not documented by default during the TIPS procedure in our hospital. Therefore, it was not possible to retrospectively assess the number of puncture attempts. This restricts the comparability to the described 3D guidance techniques. The low number of included patients and the comparison to a previous cohort from our hospital limits the transferability of our results. Another limitation is the lack of long-term shunt patency and overall survival data. Larger cohort studies with long-term follow-up examinations will be required to assess if guidance techniques have a survival benefit for the patients.

## Conclusion

TIPS placement using the 3D/2D image registered 3D-VM is technically feasible, successful, fast and safe. It allows for an efficient use of pre-procedural CE-MDCT information during the procedure in real-time. 3D/2D image registration of the 3D-VM requires only two fluoroscopic images with minimal radiation exposure. It is a promising tool for guidance of the hepatic vein catherization and the portal vein puncture with the potential to reduce radiation exposure.

## Data Availability

All data generated or analyzed during this study are included in this published article [and its supplementary information files].

## Abbreviations

2D: Two-dimensional
3D: Three-dimensional
3D-VM: Three-dimensional vascular map
BC: Budd-Chiari syndrome
BMI: Body-Mass-Index
CACT: C-arm computed tomography
CE: Contrast enhanced
CE-CACT: Contrast-enhanced C-Arm-Computed Tomography
DAP: Dose area product
DSA: Digital subtraction angiography
EASL: European Association of the Study of the Liver
FS: Fluoroscopy
FT: Fluoroscopy time
Gy cm^2^: Gray-centimeters squared
HBV: Hepatitis virus B
HC: Hemochromatosis
HCC: Hepatocellular carcinoma
HCV: Hepatitis virus C
HVCT: Hepatic vein catheterization Time
hv: Hepatic veins
IVC: Inferior vena cava
kg: Kilogram
m^2^: Square meter
MDCT: Multi-detector-computed tomography
MDCTA: Contrast-enhanced multi-detector-computed tomographic angiography
MELD: Model of endstaged liver disease
min: Minutes
mmHg: Milliliter of mercury
MRI: Magnetic resonance imaging
n.a.: Not available
NASH: Non-alcoholic steatohepatitis
OPT: Overall procedural time
PACS: Picture archive and communication system
post-LTx: Cirrhosis after orthotopic liver transplantation
PSG: Portosystemic gradient
pvb: Portal vein branches
PVP: Portal venous pressure
PT: Puncture time
TIPS: Transjugular intrahepatic portosystemic shunt
vnp: Virtual needle path
